# Integrating transcriptome and metabolome reveals molecular networks involved in genetic and environmental variation in tobacco

**DOI:** 10.1093/dnares/dsaa006

**Published:** 2020-04-23

**Authors:** Pingping Liu, Jie Luo, Qingxia Zheng, Qiansi Chen, Niu Zhai, Shengchun Xu, Yalong Xu, Lifeng Jin, Guoyun Xu, Xin Lu, Guowang Xu, Gangjun Wang, Jianfeng Shao, Hai‐Ming Xu, Peijian Cao, Huina Zhou, Xusheng Wang

**Affiliations:** 1 China Tobacco Gene Research Center, Zhengzhou Tobacco Research Institute of CNTC, Zhengzhou 450001, China; 2 Central Laboratory of Zhejiang Academy of Agricultural Sciences, Zhejiang Academy of Agricultural Sciences, Hangzhou 310021, China; 3 Key Laboratory of Separation Science for Analytical Chemistry, Dalian Institute of Chemical Physics, Chinese Academy of Sciences, Dalian 116023, China; 4 Institute of Crop Science and Institute of Bioinformatics, College of Agriculture and Biotechnology, Zhejiang University, Hangzhou 310058, China; 5 Department of Biology, University of North Dakota, Grand Forks, ND, 58202, USA

**Keywords:** tobacco, omics integration, gene regulatory networks, co-expression module, molecular network

## Abstract

Tobacco (*Nicotiana tabacum*) is one of the most widely cultivated commercial non-food crops with significant social and economic impacts. Here we profiled transcriptome and metabolome from 54 tobacco samples (2–3 replicates; *n *=* *151 in total) collected from three varieties (i.e. genetic factor), three locations (i.e. environmental factor), and six developmental stages (i.e. developmental process). We identified 3,405 differentially expressed (DE) genes (DEGs) and 371 DE metabolites, respectively. We used quantitative real-time PCR to validate 20 DEGs, and confirmed 18/20 (90%) DEGs between three locations and 16/20 (80%) with the same trend across developmental stages. We then constructed nine co-expression gene modules and four co-expression metabolite modules , and defined seven *de novo* regulatory networks, including nicotine- and carotenoid-related regulatory networks. A novel two-way Pearson correlation approach was further proposed to integrate co-expression gene and metabolite modules to identify joint gene–metabolite relations. Finally, we further integrated DE and network results to prioritize genes by its functional importance and identified a top-ranked novel gene, LOC107773232, as a potential regulator involved in the carotenoid metabolism pathway. Thus, the results and systems-biology approaches provide a new avenue to understand the molecular mechanisms underlying complex genetic and environmental perturbations in tobacco.

## 1. Introduction

Tobacco (*Nicotiana tabacum*) has great social and economic impacts on public health worldwide.^1^ One way to mitigate the impacts is to improve the tobacco quality, including both agronomic and quality traits, such as reduction of alkaloid level.^2^ Previous studies have revealed that most agronomic and quality traits are primarily influenced by both genetic and environmental factors, as well as developmental processes.^3^ However, molecular networks by which genetic and environmental factors influence these traits remain elusive.

One strategy to probe the molecular network(s) is a systematical characterization of molecular compositions (i.e. gene, protein, and metabolite) under genetic, environmental, and developmental perturbations. With the advent of high-throughput omics technologies, we are now capable of identifying and quantifying transcriptome, proteome, and metabolome by next-generation sequencing and high-resolution mass spectrometer (MS). In tobacco, whole-genome and RNA sequencing identified ∼90,000 genes and over 134,000 transcripts.^4–6^ Recently, several research groups analysed tobacco transcriptome to understand transcriptional regulation involved in various agronomic and quality traits, such as response to premature senescence^7^ and response to topping.^8^

Tobacco is also a secondary metabolite-rich crop, with more than 4,000 different metabolites in its leaves.^9^^,^^10^ Many agronomic traits have been associated with metabolites in plant.^9^^,^^11^^,^^12^ Recently, researchers used both targeted and untargeted metabolomics technologies to characterize metabolic responses to environmental factors and developmental processes in tobacco.^13^^,^^14^ Although these studies provided a list of altered metabolites under perturbations, little is known about the underlying molecular networks and interaction between metabolites with genes underlying agronomic and quality traits in tobacco.

While analysis of a single omics data (e.g. transcriptome or metabolome) provides biological understanding at a specific molecular layer, many agronomic and quality traits involve complex crosstalk between different molecular layers, such as genome, proteome, and metabolome. An integrative analysis of multiple layers of molecular data helps to discover and elucidate underlying molecular mechanisms of complex traits. For example, we recently integrated transcriptomics and metabolomics data to identify molecular networks in response to cold stress in tobacco.^15^

Several statistical algorithms have been proposed to tackle multi-omics data, including unsupervised, and supervised data integration methods.^16^ Unsupervised data integration refers to the cluster of methods that draw an inference from input datasets without labelled response variables. The two most widely used approaches are partial least squares (PLS) ^17^ and canonical correlation analysis (CCA).^18^ However, the CCA method is a linear technique in which each component is constructed based on a linear combination of variables. Although PLS assumes a non-linear relationship between different sets of data, the interpretation of the results is not straightforward. Compared with the unsupervised data integration methods, the supervised methods consider sample information (e.g. disease or normal), and then performs data integration with sophisticated statistical or machine learning methods. Representative methods include network-based, multi-kernel and multi-step-based methods. However, the main limitation of the supervised methods is that the learning process heavily depends on the quality of the training omics datasets.

In this study, we profile both transcriptome and metabolome from 54 tobacco samples collected from six developmental stages of three widely planted varieties (K326, Hongda, and Zhongyan100) planted in three most tobacco productive regions (Henan, Guizhou, and Yunnan) in China. We identify differentially expressed genes (DEGs) and metabolites (DEMs), and construct co-expression modules for both DEGs and DEMs, and validate them by quantitative real-time PCR (qRT-PCR). We further define gene regulatory networks (GRNs) for co-expression gene modules. We also propose a novel approach to integrate transcript and metabolite co-expression modules to identify joint gene-metabolite relations. Finally, we integrate DE and network results to identify a novel gene, LOC107773232, as a potential regulator in the carotenoid metabolism pathway.

## 2. Materials and methods

### 2.1. Tobacco samples

A total of 54 tobacco samples were used, including three varieties (Hongda, k326, and Zhongyan100) planted in three locations (Guizhou, Henan, and Yunnan) in China across six developmental stages, each with 2–3 biological replicates (*n *=* *151). The six developmental stages include vigorous growth (S1), budding (S2), fully blooming (S3), maturity of lower leaves (S4), pre-maturity of middle leaves (S5), and maturity of middle leaves (S6). The six developmental stages were determined by morphologic features of the tobacco. Vigorous growth (S1) is an important stage at which tobacco grows rapidly, typically during the period of ∼30–60 days after transplanting. We collected samples at 50 days after transplanting. Budding (S2) is a stage of ∼50% plants with buds. Fully blooming (S3) is a stage of >50% plants in blossom. The maturity of lower leaves (S4) is a stage at the beginning of lower leaves turning yellow. The pre-maturity of middle leaves (S5) is at the stage of 10 days after S4. The maturity of middle leaves (S6) is the time that parts of middle leaves turn yellow.

### 2.2. RNA isolation, microarray analysis, and qRT-PCR

The total RNA of each sample was extracted from fresh leaves using the Qiagen RNAeasy Mini Kit (Qiagen) on leaves of three independent plants. The RNA quality was verified using a Bioanalyzer (Agilent Technologies, Santa Clara, CA, USA), and samples with an RNA integrity number (RIN) value above 7.5 were selected. A total of 5.5 μg of the ampliﬁed products were used.

The labelled cDNAs 5.2 µg were dissolved in 160 μl of hybridization mix solution and then denatured at 99°C for 5 min. The mixed hybridization buffer was loaded into a microarray, and performed in a hybridization oven (Affymetrix) at 45°C for 16 h. The array is a customized chip, including 81,251 probe sets, corresponding to 77,924 probe sets.

For qRT-PCR, total RNAs were extracted from tobacco samples from three locations and six development stages using a EASYspin plus plant RNA isolation kit (Aidlab, China). After evaluation of RNA concentration by a Nanodrop 2000 instrument (Thermo), cDNA synthesis was performed with 1 µg RNA of each sample per 20 µl reaction using a Transcriptor First Strand cDNA Synthesis Kit (Roche), according to the manufacturer’s protocol. Quantitative PCR reactions were prepared in triplicate using 2 µl of 10 times diluted cDNA, 0.5 µl of each primer in 10 mM, and 10 µl of 2×SYBR master buffer (Roche) in a reaction volume of 20 µl. The qPCR reactions were performed on a LightCycler^®^ 96 machine (Roche) using the following program: (i) 95°C for 10 min, (ii) 95°C for 10 s, (iii) 50–60°C for 15 s depending on primer Tm, (iv) 72°C for 15 s for fluorescence measurement, (v) go to step (ii) and repeat 40 cycles, (vi) additional final cycle at 95°C for 5 s, 65°C for 1 min, and 97°C for 1 s at a continuous fluorescence acquisition mode to analyse the specific amplification of targets, and (vii) 40°C for 10 s. Relative transcription level of each target gene was calculated using a 2-ΔCt formula, in which ΔCt was the difference between Ct values of target gene to reference gene. Tobacco EF1α gene was used as reference. All primers used for RT-qPCR analyses are listed in Supplementary Table S9.

### 2.3. Untargeted metabolomics using gas chromatography-mass spectrometry, liquid chromatography-mass spectrometry , and capillary electrophoresis-mass spectrometry

We performed untargeted metabolome profiling using three platforms: gas chromatography-mass spectrometry (GC-MS), liquid chromatography-mass spectrometry (LC-MS/MS), and capillary electrophoresis-mass spectrometry (CE-MS). For the GC-MS, the freeze-dried tissues were ground into powder, and 20 mg was extracted in 1.5 ml solution of isopropanol/acetonitrile/water (3/3/2, v/v/v) with 15 μl (2 mg/ml) tridecanoic acid as an internal standard. After 1 h extraction with sonication, 500 μl of supernatant was collected by 10 min centrifugation (150,00 rpm). The supernatant was dried in a vacuum condenser prior to derivatization with pyridine solution of methoxamine hydrochloride (37°C, 90 min), and MSTFA (60°C, 60 min). The metabolomic analysis was performed on an Agilent 5975C according to a pseudotargeted method.^19^ Briefly, the Agilent DB-5MS column (0.25 μm, 0.25 mm × 30 m) was used, and the injection volume was 1 μl with a 10:1 split ratio. The detector voltage was maintained at 1.2 kV, and the electron impact model was selected to achieve ionization of the metabolites at 70 eV.

For the LC-Q-TOF/MS, 20 mg of lyophilized tobacco leaf powder was extracted with 1.5 ml of the extraction solution [5 μg/ml umbelliferone (internal standard) in methanol/water (3:1, v/v)]. After 1 h ultrasonication, insoluble material was removed by centrifugation at 12,000×*g* for 10 min, and a total of 800 μl of the supernatant was transferred to a glass vial prior to injection into the instrument. The LC-MS analysis was performed on an Agilent 1290 Series UHPLC system coupled to an Agilent 6540 TOF/MS instrument with a Dual AJS ESI source. Chromatographic separation was achieved using an Agilent Zorbax SB-C18 (RRHD, 2.1 mm × 100 mm, 1.8 μm particle) analytical column operated at 60°C. Mass spectrometric analysis was performed in positive mode with full scan mode from 50 to 1,000 *m*/*z*.

For the CE-MS, fresh samples were used and analysed by an Agilent G7100A CE system coupled to a G6224A TOF/MS according to the method described previously.^13^

### 2.4. Analysis of DE genes and metabolites

DE genes and metabolites were analysed using a full analysis of variance (ANOVA) model as follows:

$$y_{\mathrm{ijkl}}=\mu +C_{i}+\;l_{j}+\;S_{k}+\;{C\times l}_{ij}+\;{C\times S}_{ik}+\;{l\times S}_{jk}+{C\times l\times S}_{\mathrm{ijk}}+\varepsilon_{\mathrm{ijkl}}$$

where $y_{\mathrm{ijkl}}$ is log2 transformed expression level of each gene or metabolite, µ is the grand mean, three single factors [i.e. variety ($C_{i}$), location ($l_{j}$), and stage ($S_{k}$]), three double interactions (variety × location, variety × stage, and location × stage), and one triple interaction (i.e. variety × location × stage). $\varepsilon_{\mathrm{ijkl}}$ is the model residual. This analysis was performed using R package (version 3.2.5).

For each gene or metabolite, the *P*-value was calculated using the ANOVA method, and multiple corrections were performed using the Benjamini and Hochberg (BH) method.^20^ We applied an adjusted *P*-value of 0.01 as a threshold to define DEGs or metabolites.

### 2.5. Principal component analysis and hierarchical clustering

Principal component analysis (PCA) was used to visualize the differences among samples. All gene and metabolite abundance were used as features of PCA. The pairwise Euclidean distance between features was calculated. PCA was performed using the R package prcomp (version 3.4.0).^21^

The hierarchical clustering of DE genes and metabolites was performed to determine the differences among diverse groups. Hierarchical clustering was carried out using the heatmap.2 function in R statistical analysis package. The clustering for heatmap.2 was obtained with Ward’s linkage and Euclidean distance. Disease groups were clustered and visualized with heat map based on the scaled expression values.

### 2.6. Pathway enrichment by gene ontology, KEGG, and hallmark databases

Pathway enrichment analysis was carried out to infer functional groups of genes and metabolites that were enriched in a given dataset. The analysis was performed using Fisher’s exact test^22^ with the BH correction for multiple testing (FDR). Enriched pathways with FDR < 5% were considered statistically significant.

For gene functional enrichment, we used gene ontology (GO) database downloaded from the plantGSEA databases (http://structuralbiology.cau.edu.cn/PlantGSEA). For metabolite enrichment analysis, we used 118 curated pathways, including amino acid metabolism, carbohydrate metabolism, carotenoid metabolism, chlorophyll metabolism, glycolysis/glucolysis, lipid metabolism, nicotine metabolism, phenylpropanoids, and flavonoid metabolism, polyamine biosynthesis, sterol metabolism, and tricarboxylic acid cycle (Supplementary Table S8).

### 2.7. Genome-wide co-expression analysis for DEGs and DEMs

We constructed weighted gene co-expression networks based on DEGs and metabolites using the WGCNA package in R. The product was a weighted adjacency matrix that provided continuous connection strength ([0, 1]) based on the *β* parameter for each condition to meet the scale-free topology criterion. The concept of the scale-free network has emerged as a powerful paradigm in the study of network biology. Most biological networks, such as metabolic, protein, and gene interaction networks, have been reported to exhibit scale-free behaviour based on the analysis of the distribution of the number of connections of the network nodes.^23^ A scale-free network is one whose majority nodes has only a few connections to other nodes, whereas some nodes (hubs) are connected to many other nodes in the network. The number of connections each node has is called its degree. If we represent the degree distribution of a scale-free network in a logarithmic scale, we can see how it fits with a line (they fit a power-law), having a small number of nodes with high degree (the hubs), and a large number of nodes with a low degree. Subsequently, the co-expression matrix and the topological overlap matrix (TOM) were constructed. For TOM, we assessed the interconnectedness of two genes by the degree of their shared neighbours across the global network. We detected the gene modules by average linkage hierarchical clustering for each group. The intra-modular connectivity of each gene was also computed using the intra-modular connectivity function in R. The module eigengene (ME) is the first principal component of a given module, and it was used to evaluate the module membership, which assesses the importance of genes in the network. We used the *β* power parameter of 7 for both gene and metabolite co-expression analyses.

### 2.8. Regulatory network prediction using ARACNe-AP

Transcriptional regulatory networks were predicted for each co-expression modules using ARACNe-AP.^24^ For each module, the input to ARACNe-AP consisted of a matrix containing the gene expression data of each co-expression module and a list of genes in the pathway. We used 422 genes from eight curated pathways (Supplementary Table S8), including aspartate oxidase (AO), NtADC, and ornithine decarboxylase (ODC). A *P*-value of 1.0 × 10^−8^ was used as a threshold for mutual information. Predicted networks were visualized in Cytoscape^25^ within ARACNe.^26^

### 2.9. Integration of transcriptomic and metabolomic data using two-way correlation

To integrate transcriptomic and metabolomics data, we performed two-way Pearson correlations: (i) between each metabolite and the eigenvector from nine co-expression gene modules; (ii) between each gene and the eigenvector from four co-expression metabolite modules. A *P*-value < 0.01 was used to identify statistically significant correlation.

### 2.10. Ranking genes with Fisher’s combined probability test

To rank genes by its functional importance, we calculated a combined score using Fisher’s combined probability test.^27^ For each gene, we first computed five individual *P*-values as follows:

1. *P*-value of the correlation between the expression of each gene and its associated co-expression module. We use the module membership (kME) for each co-expression module, which was generated by the WGCNA program;
2. *P*-value of differential gene expression between three locations;
3. *P*-value of differential gene expression between three varieties;
4. *P*-value of differential gene expression between six developmental stages;
5. *P*-value of the correlation between the expression of each gene and the best-correlated metabolite module (i.e. kME).

Then, we generated a combined *P*-value for each gene using Fisher’s method, which combines extreme value probabilities from each test into one test statistic ($x^{2}$):

$$x_{2k}^{2}\;∼-2\sum_{i=1}^{k}\ln(p_{i})$$

where $p_{i}$ is each individual *P*-value. A chi-square test is used to calculate the significance level of the combined analysis, in which the degree of freedom is 2*k*. The final combined *P*-value is transformed into a final score of –log_10_(combined *P*-value).

## 3. Results

### 3.1. Global transcriptome and metabolome profiling of tobacco leaves

**Figure 1:**
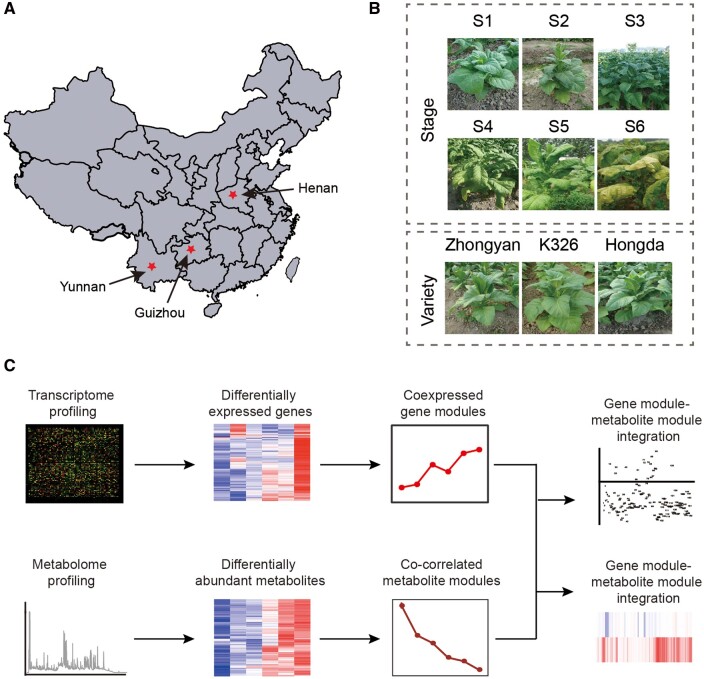
Experimental design and data analysis in this study. (A) Geographic map of part of China showing locations of the three most tobacco productive areas in China: Henan, Yunnan, and Guizhou. (B) Samples of tobacco leaves collected from three varieties (K326, Hongda, and Zhongyan100), three locations (Henan, Yunnan, and Guizhou), and six developmental stages (S1: vigorous growth, S2: budding, S3: full-bloom, S4: the maturity of lower leaves, S5: pre-maturity of middle leaves, and S6: maturity of middle leaves). (C) Schematic diagram showing data generation and analysis in this study. Four phases of the analysis are performed: (1) profiling transcriptomic and metabolomic data; (2) detecting differentially expressed transcripts and metabolites; (3) identifying genome-wide transcript and metabolite co-expression modules; and (4) integrating transcript and metabolite co-expression modules.

To explore the effects of genetic and environmental factors as well as developmental processes on agronomic and quality traits, we collected 54 tobacco leaf samples (Supplementary Table S1), composed of six developmental stages of three varieties planted in three regions in China. The three regions, Yunnan, Guizhou, and Henan, are the most tobacco productive areas in China (Fig. 1A). Yunnan is located in southwest China, has the highest sun exposure time during the vigorous growth period, and less temperature and rainfall (Supplementary Fig. S1). Guizhou lies in the northeast of Yunnan, a subtropical plateau with median temperature and abundant rainfall, but less sun exposure time compared with Yunnan and Henan (Supplementary Fig. S1). Henan is located in the central plain of China where the temperature is considerably high and has mediate sun exposure time and rainfall (Supplementary Fig. S1). The three regions represent three different flavour styles of the flue-cured tobacco: light, strong, and intermediate flavours. The three varieties, K326, Hongda, and Zhongyan100, are accompanied by substantially differ in agronomic, quality, and physiological traits. For example, K326 has higher alkaloids than Zhongyan 100 and Hongda.^28^^,^^29^ The six major developmental stages include vigorous growth (S1), budding (S2), full-bloom (S3), maturity of lower leaves (S4), pre-maturity of middle leaves (S5), and maturity of middle leaves (S6) (Fig. 1B). In this study, we performed four-phase analyses (Fig. 1C): (i) profiling transcriptomic and metabolomic data from the 54 leaf samples; (ii) detecting differentially expressed transcripts and metabolites under genetic (i.e. three varieties) and environmental perturbations (i.e. three regions), and six developmental stages; (iii) identifying genome-wide co-expression gene and metabolite modules and constructing regulatory networks for co-expression gene modules; and (iv) integrating transcript and metabolite co-expression modules to identify joint gene–metabolite relations and potential candidate regulators.

**Figure 2:**
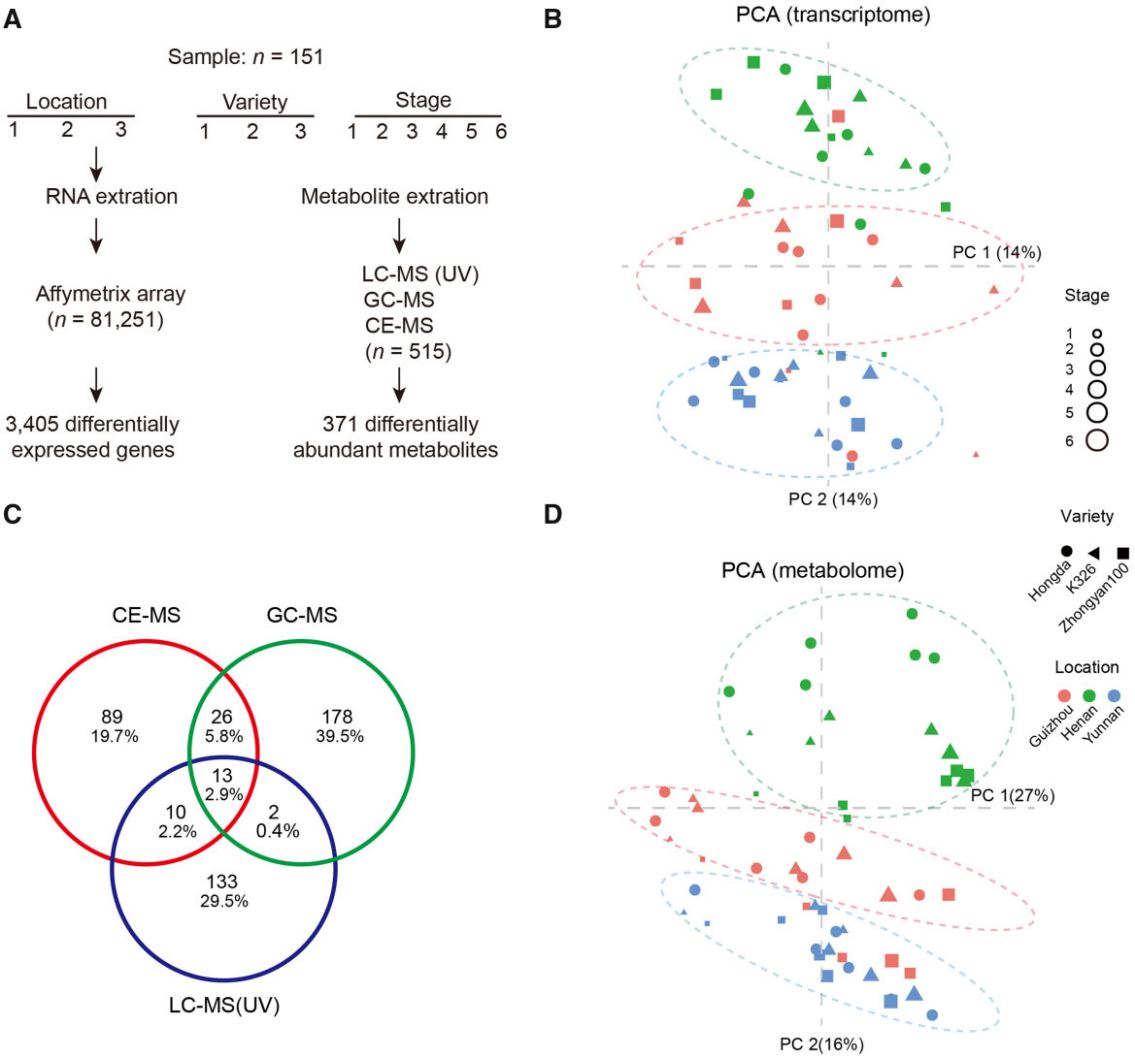
Profiling of transcriptome and metabolome of 54 tobacco samples. (A) Flowchart showing data processing of transcriptomic and metabolomics data. A total of 151 samples were used for transcriptome and metabolome profiling, resulting in 3,405 differentially expressed genes, and 371 differentially expressed metabolites, respectively. (B) PCA plots of the 54 samples (∼3 replicates per sample) using transcriptomic data. (C) Venn diagram showing metabolites detected by CE-MS, GC-MS, and LC-MS. (D) PCA plots of the samples using metabolomic data.

Transcriptomic data of fresh leaves were profiled using a customized Affymetrix microarray. A total of 81,251 probe sets were used to measure the expression of transcripts (Fig. 2A). We annotated these probe sets by mapping the sequences to the genome of K326, a tobacco reference genome.^4^ A total of 77,924 probe sets can be mapped to the tobacco genome, and are used for the subsequent analysis. PCA showed an apparent clustering of three locations by PC2 (Fig. 2B), and good separation of developmental stages by PC1 (Supplementary Fig. S2), indicating a high quality of the RNA samples.

We also profiled metabolome of leaves using three mass spectrometry platforms, including GC-MS, LC-MS, and CE-MS. Leaves were freeze-dried for GC-MS and LC-MS and fresh for CE-MS. We identified and quantified a total of 2,075 unique features and annotated 759 metabolites. After the removal of the duplicates, 561 metabolites with unique structure annotations were used for the subsequent analysis (Supplementary Table S2). The 515 metabolites include 219 metabolites detected by GC-MS, 158 metabolites by LC-MS, and 138 by CE-MS, respectively (Fig. 2C). As expected, polar metabolites (e.g. amino acids, sugar, and nucleic acids) were detected by GC-MS and CE-MS, whereas second-metabolism compounds (e.g. flavonoids and terpenes) were identified by LC-MS. Only 51 out of 561 metabolites were detected by two or three platforms, indicating three platforms complement one another in metabolomic profiling. Similar to transcriptomic analysis, PCA showed a separation for locations and developmental stages by the first two principal components (Fig. 2D).

### 3.2. Differentially expressed gene analysis 

**Figure 3:**
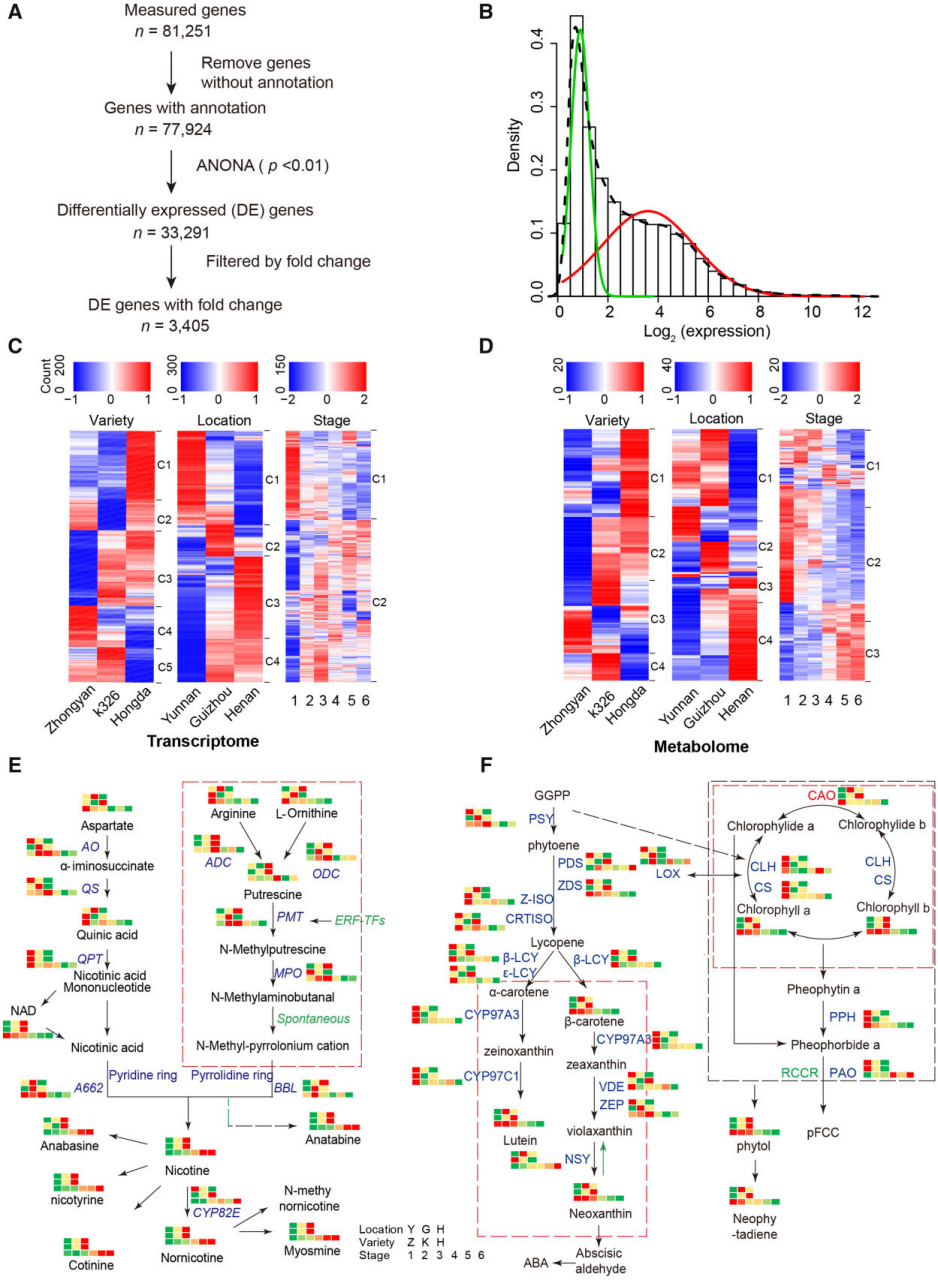
Analyses of differentially expressed genes and metabolites. (A) Diagram showing the identification of differentially expressed genes. (B) Detection of highly variable genes. The variability of gene expression is measured by the coefficient of variation (CV) across 151 samples. The distributions of CV were fitted by two normal distributions using the EM algorithm. (C) Heat map of differentially expressed genes showing changes and expression patterns (clusters) in expression between three varieties (left panel), three locations (middle panel), and six developmental stages (right panel). A total of 941, 2,763, and 2,838 differentially expressed genes between three varieties, three locations, and six stages with a cutoff of FDR < 0.01, respectively. Those differentially expressed genes can be found in Supplementary Table S3A. (D) Heat map of differentially expressed metabolites showing changes and expression patterns (clusters) in metabolite abundance. A total of 192, 291, and 297 differentially expressed genes between three varieties, three locations, and six stages with a cutoff of FDR < 0.01, respectively. Those differentially expressed genes can be found in Supplementary Table S3B. (E) Nicotine synthesis pathway labelled with changes in gene and metabolite abundances. Expression levels of each gene across 151 samples were scaled into a relative expression scale using *z*-score transformation. Relative expression was represented using a red–yellow–green colour scale: three locations (top layer), three varieties (middle layer), and six stages (bottom layer). Red represents up-regulation and green indicates down-regulation. (F) Carotenoid metabolism pathway labelled with changes in gene and metabolite abundances. The same data transformation and colour scheme are used as panel E. Color figures are available at *DNARES* online.

To identify genes influenced by genetic and environmental perturbations, and developmental processes, we performed differential expression analysis for both transcriptomic data (Fig. 3A). First, we removed low-expressed transcripts by decomposing all transcripts with high and low expression using the expectation-maximization (EM) algorithm, retaining 43,675 out of 77,924 transcripts for subsequent analyses (Fig. 3B). By using ANOVA and multiple test correction, we detected 2,764 DEGs between three varieties, 24,705 DEGs between three locations, and 21,584 DEGs among six stages at adjusted *P*-value <0.01. To detect genes with relatively large variation, we applied at least two S.D.^30^ of fold change to each DEG. We finally identified 3,405 DEGs, including 941 DEGs between three varieties, 2,838 DEGs between six growth stages, and 2,763 DEGs between locations (Supplementary Table S3A and Fig. 3C). The vast majority (2,402/3,388; 70.54%) of DEGs is shared between locations and developmental stages, suggesting that a potential common molecular mechanism is involved in response to environmental perturbation and developmental processes (Supplementary Fig. S3A).

As many DEGs are novel, we next assessed changes in mRNA expression of 20 selected genes between three locations and six developmental stages using qRT-PCR. Of the 20 selected DEGs, 18/20 (90%) were validated as DEGs between the three locations (Supplementary Fig. S4A), and 16/20 (80%) showed the same trend as measured by the Microarray (Supplementary Fig. S4B). Thus, the results largely support the reproducibility of DE genes from the microarray analysis. For example, both Microarray and qPCR validated that a chlorophyll a–b binding gene, CAB16, showed a marked decrease in Henan compared with Yunnan and Guizhou (Supplementary Fig. S4C), and a decrease in expression along the six developmental stages (Supplementary Fig. S4D). The result is consistent with the fact that CAB16 serves as the major pigment binding protein in functional photosystem II reflecting the degradation of chloroplast along the developmental stages and the climatic factors as Guizhou typically receives more rainfall slowing the leaf senescence.

### 3.3. Functional enrichment analysis of DEGs and DEMs

To further understand the biological function of DEGs in response to genetic and environmental perturbations as well as developmental processes, we performed GO enrichment analysis using Fisher’s exact test with BH multiple testing correction. To do this, we further divided the DEGs into different categories with respect to their expression patterns, resulting in five clusters of expression pattern in the three varieties, four clusters in the three locations, and two clusters in six developmental stages, respectively (Fig. 3C). The functional enrichment analysis for each cluster identified 13 significant enrichments in several GO terms and pathways (Supplementary Table S6). For those DEGs of the three varieties, only cluster 3 was shown to be significantly enriched in photosynthesis (GO: 0009765; adjusted *P*-value of 0.03). In contrast, all four clusters of locational DEGs exhibited a significant enrichment. The cluster 1 also showed significant enrichment in photosynthesis (GO: 0019684; adjusted *P*-value of 2.00 × 10^−2^), indicating that a subset of genes is shared a similar molecular process in photosynthesis between variety and location. Both clusters 2 and 3 share two same significant enrichments: (i) response to stimuli, including light intensity (GO: 0009642; adjusted *P*-value of 5.36 × 10^−3^), oxidative stress (GO : 0006979; adjusted *P*-value of 3.40 × 10^−2^), and temperature (GO : 0009266; adjusted *P*-value of 4.68 × 10^−3^); (ii) cellular metabolic process (GO : 0044260; adjusted *P*-value of 3.80 × 10^−2^). The cluster 4 displays enrichment in immune system process (GO : 0002252; adjusted *P*-value of 3.80 × 10^−2^), regulation of biological process (GO : 0043069; adjusted *P*-value of 2.54 × 10^−2^), and response to stress (GO : 0002679; adjusted *P*-value of 1.32 × 10^−2^). For DEGs between six developmental stages, we found that cluster 1 shows enrichments in developmental process (GO : 0048731; adjusted *P*-value of 2.39 × 10^−2^), and metabolic process (GO : 0009820; adjusted *P*-value of 3.36 × 10^−9^), whereas the cluster 2 enriched in response to stimulus (GO : 0009605; adjusted *P*-value of 8.28 × 10^−3^), and regulation of biological process (GO : 0043069; adjusted *P*-value of 6.93 × 10^−3^).

Similarly, we identified 361 DEMs with adjusted *P*-value < 0.01 (Supplementary Table S3B), including 192 DEMs between three varieties, 291 DEMs between three locations, and 297 DEMs between six developmental stages (Fig. 3D). The vast majority of the DEMs (134/371; 36.12%) showed changes in all three comparisons (Supplementary Fig. S2B). Functional enrichment analysis for DEMs using Metabolite Set Enrichment Analysis in the MetaboAnalyst tool showed only three significant enrichments in the cluster 3 of DEMs from the developmental stage, including glycine and serine metabolism (adjusted *P*-value of 0.01), methionine metabolism (adjusted *P*-value of 0.02), and aspartate metabolism (adjusted *P*-value of 0.04).

We next projected abundance changes at both transcript and metabolite levels to the two most important pathways involved in tobacco quality traits: nicotine synthesis pathway and carotenoid synthesis pathway (Fig. 3E and F). Nicotine is a secondary alkaloid synthesized primarily in roots but accumulated in leaves. Nicotine synthesis is through a pathway of the pyridine and pyrrolidine rings in which several key genes and metabolites are involved, referred to as the nicotine synthesis pathway.^31^ The pyrrolidine ring pathway starts with putrescine, which can be formed directly from l-ornithine by ODC and/or synthesized indirectly from arginine by arginine decarboxylase (ADC). Putrescine is converted to *N*-methylputrescine by putrescine *N*-methyltransferase. *N*-methylputrescine is then oxidized by *N*-methylputrescine oxidase, and cyclized to form the pyrrolidine ring. On the other hand, the pyridine ring pathway is composed of several genes, including AO, quinolinic acid synthase, and quinolinic acid phosphoribosyl transferase.^4^^,^^6^

While most alkaloids are thought to be synthesized in roots and accumulated in leaves, most metabolites in the nicotine synthesis pathway in leaves showed up-regulation during the development stages (Fig. 3E). Nicotine and other five major tobacco alkaloids (i.e. anabasine, nicotyrine, cotinine, nornicotine, and myosmine) show relatively low at both transcript and metabolite abundance during the early stages and reach their maximum around the stages of 5 and 6. In general, genes show an earlier response than metabolites. For example, the level of gene AO raised for 4.35-fold from stage 1 to 2 and slightly decreased after the stage 2, whereas the level of metabolite nicotine in the pathway gradually increase for 2.73-fold from stage 1 to 6 (Supplementary Fig. S5A and B). Because gene expression is transient and we measured the expression in leaves instead of the roots, most genes response in early developmental stages. When comparing metabolite abundance between varieties, we found that alkaloids in Hongda showed a higher level than that in Zhongyan and K326. For three locations, the abundance of alkaloids in Henan is higher than that in Yunnan and Guizhou.

We also examined abundance changes at transcript and metabolite levels in the carotenoid synthesis pathway. Carotenoids are the most common group of pigments observed in plant, playing essential roles in development, photosynthesis, and membrane stability. The pathway starts with a head-to-head coupling of two molecules of geranylgeranylpyrophosphate (GGPP) to yield colourless phytoene by phytoene synthase,^30^ with prephytoene diphosphate as an intermediate. Subsequently, four additional double bonds are introduced by desaturases producing the coloured carotenes phytofluene, carotene, neurosporene, and lycopene. Lycopene is cyclized twice by two individual cyclases, yielding α- and β-carotene, which are subsequently processed to different xanthophylls, such as lutein, violaxanthin, and zeaxanthin. We found that genes and metabolites showed significant decrease along the developmental stages (Fig. 3F). For example, the level of gene CYP97A3 and metabolite beta-carotene dropped for 1.55-fold and 2.88-fold from stages 1 to 6, respectively (Supplementary Fig. S5C and D). This observation suggests that the photosynthetic capacity decreased, and chlorophylls started degradation when leaves became mature. Among three varieties, Hongda showed the highest abundance level compared with the other two varieties (i.e. K326 and Zhongyan100). By comparing three locations, Henan exhibited the lowest level of gene and metabolite abundance, whereas Yunnan showed the highest level (Supplementary Fig. S5C).

### 3.4. Construction of co-expression gene and metabolite modules

**Figure 4:**
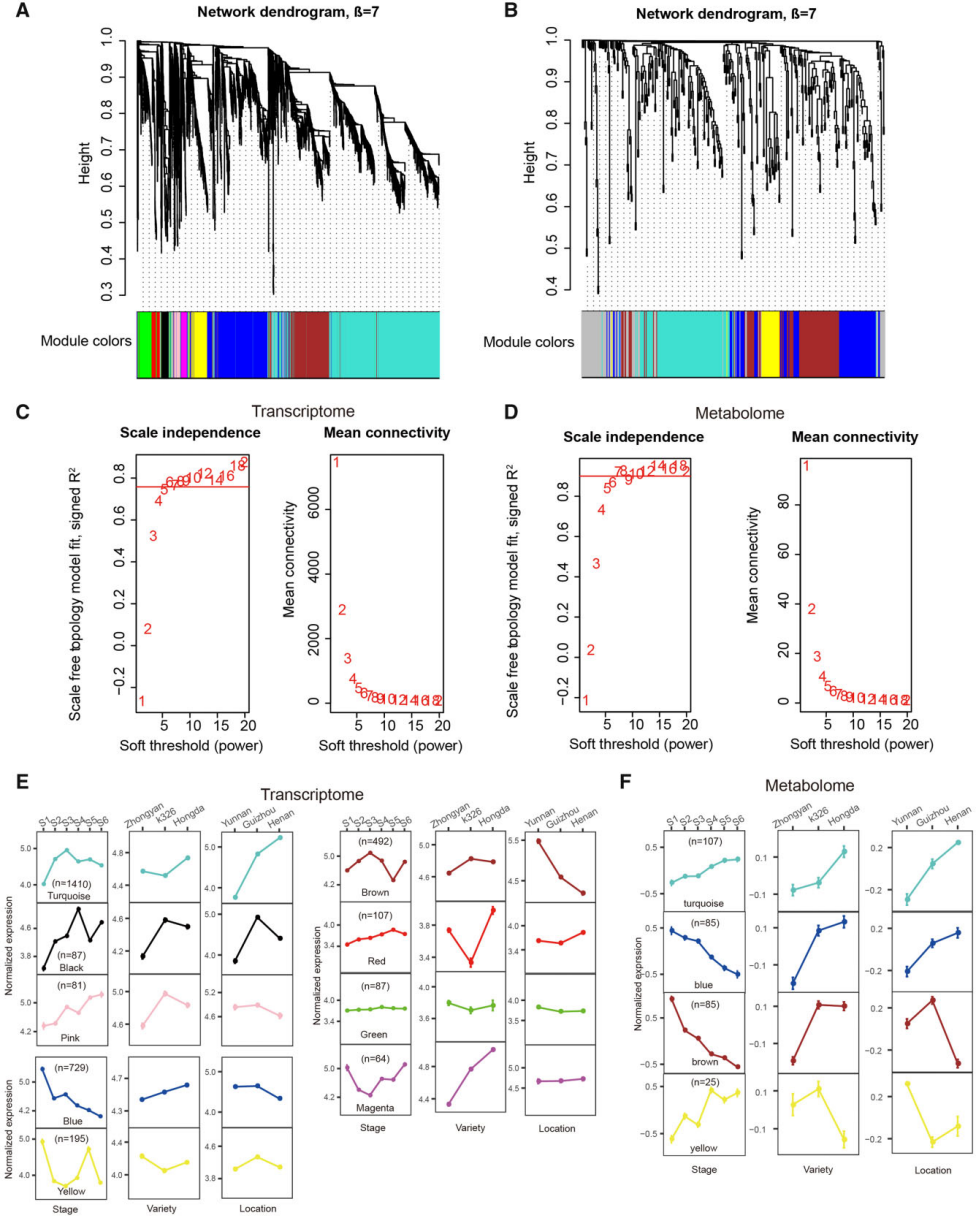
Co-expression analyses of transcriptomic and metabolomic data. (A–B) Cluster dendrogram and module assignment for modules from WGCNA. Genes were clustered based on a dissimilarity measure. The branches correspond to modules of highly interconnected groups of genes. Colours in the horizontal bar represent the modules. A total of nine gene modules and four metabolite modules were identified by WGCNA from transcriptomic and metabolic data, respectively. Genes that did not belong to any modules were described in the grey modules, which were discarded in the subsequent study. (C–D) Selection of the soft-thresholding powers for scale-free co-expression network. The left panel showed the scale-free fit index versus soft-thresholding power. The right panel displayed the mean connectivity versus soft-thresholding power. Power 7 for both transcriptome and metabolome was used. (E–F) Average co-expression pattern of gene and metabolite modules. The error bar represents S.E.M.. For each module, three conditions are shown from left to right: stage, variety, and location. Color figures are available at *DNARES* online.

To further gain insights into the expression and functional organization of the transcriptome and metabolome, we constructed gene and metabolite co-expression modules for 3,405 DEGs and 371 DEMs, respectively (Supplementary Tables S4A and B) using WGCNA program. We identified a total of nine gene modules (Supplementary Table S5; Fig. 4A) with soft-thresholding power (β  =  7) in accordance with the scale-free topology criterion (Fig. 4C). Supplementary Table S5; Fig. 4A The module size (i.e. the total number of genes in a module) varies significantly, ranging from 64 genes in the magenta module to 1,410 genes in the turquoise module (Supplementary Table S5). To investigate changes in expression pattern with respect to three varieties, three locations, and six developmental stages, we summarized the nine detected co-expression gene modules into three patterns in terms of up- or down-regulation. For example, the turquoise module shows up-regulation along the developmental stages, a higher level in expression in Hongda than K326 and Zhongyan100, and in Henan than Yunnan and Guizhou (Fig. 4E). We also examined whether any pre-defined pathways are enriched in each module. We found that genes involved in the carotenoid synthesis pathway are enriched in the blue module (*P*-value = 9.52 × 10^−3^); genes in the phenylpropanoids and flavonoid metabolism pathway are enriched in the brown module (*P*-value = 3.61 × 10^−3^); and genes in the chlorophyll metabolism (*P*-value = 1.3 × 10^−2^), nicotine metabolism (*P*-value = 4.33 × 10^−2^), and polyamine biosynthesis pathways (*P*-value = 4.33 × 10^−2^) are enriched in the turquoise module (Supplementary Table S4A).

Similarly, we also identified four metabolite co-expression modules (Supplementary Table S5; Fig. 4B) with soft-thresholding power (*β*  =  7) (Fig. 4D). The module size ranges from 25 metabolites in the yellow module to 107 in the turquoise module (Supplementary Table S5). Only two expression patterns were observed, including up-regulation and down-regulation along development stages (Fig. 4F). Metabolite enrichment analysis shows that the blue module has over-representative metabolites in both carotenoid synthesis pathway (*P*-value = 3.16 × 10^−2^) and lipid metabolism pathway (*P*-value = 1.48 × 10^−2^); the brown module has enriched metabolites in amino acid metabolism pathway (*P*-value = 6.3 × 10^−5^); and turquoise module has over-representative metabolites in both carbohydrate metabolism pathway (*P*-values = 1.18 × 10^−2^) and nicotine synthesis pathway (*P*-values = 1.53 × 10^−4^) (Supplementary Table S4B).

### 3.5. Gene regulatory networks (GRNs) in gene modules

**Figure 5:**
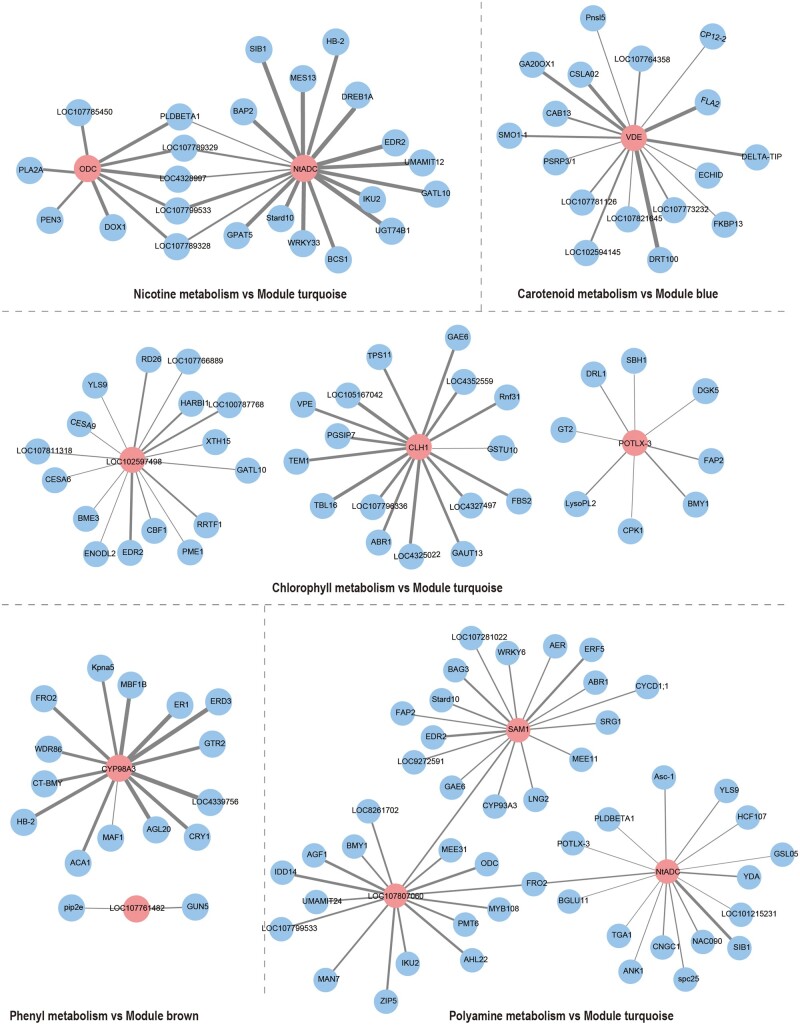
Transcriptional networks for three co-expression modules. The network was constructed with the ARACNe-AP program. Gene names in red are presented in both co-expression modules and the pathways (Supplementary Table S8). Nodes in blue are only detected in co-expression modules. The edge thickness is proportional to the correlation strength. The thicker the edge, the greater the correlation between the two genes. Color figures are available at *DNARES* online.

We next sought to derive *de novo* GRNs for co-expression gene modules using the ARACNe-AP tool,^24^ a widely used information-theoretical method for GRN reconstruction.^26^ ARACNe-AP computes expression correlations between genes and selects those statistically significant genes as edges in the GRNs.^32^ In contrast to traditional GRN construction using transcriptional factors, we used 422 genes in eight curated pathways (see Materials and methods) as regulators to identify their targets in co-expression modules. Using a stringent threshold for mutual information (*P*-value = 1.0 × 10^−8^), three co-expression modules (i.e. blue, brown, and turquoise) out of the nine co-expression modules (Fig. 5) were formed into GRNs, and we further examined biological functions of these genes.

The first regulatory network is associated with the nicotine biosynthesis pathway detected in the turquoise co-expression module. Two key nicotine biosynthesis-related genes (i.e. *ODC* and *ADC*) were found to be associated with 23 genes in a GRN. A total of nine genes are directly associated with ODC, including two genes (*PEN3* and *DOX1*), many of which are involved in defence against pathogens and herbivores, which is consistent with the function of nicotine as plant inducible defence. *PEN3* encodes the putative ATP-binding cassette (ABC) transporter, playing central components in cell wall-based defence against microbial pathogens.^33^*DOX1* encodes alpha-dioxygenase that mediates protection against oxidative stress and cell death,^34^ and is negatively associated with abscisic acid (ABA)-mediated signalling pathway.^35^ ADC is associated with 19 genes, most of which are involved in defence and protection, including two transcriptional factors (*DREB1A* and *WRKY33*), *EDR2*, and *BAP2*. The transcription factor *DREB1A* induces expression of genes involved in environmental stress tolerance in Arabidopsis.^36^^,^^37^*WRKY33* is a positive regulator of the salt stress response and ABA signalling.^38^ Similarly, EDR2 is a negative regulator of salicylic acid-mediated resistance to pathogens.^39^^,^^40^*BAP2* is a general inhibitor of programmed cell death. In addition, we also found that five genes are directly associated with both *ODC* and *NtADC*, four of which are less well annotated (*LOC107789329*, *LOC4328997*, *LOC107799533*, and *LOC107789328*), indicating that many potential genes regulating nicotine synthesis pathway are not yet fully appreciated in tobacco.

The second regulatory network is the carotenoid synthesis pathway detected in the blue co-expression module. A carotenoid related gene, *VDE*, which encodes violaxanthin de-epoxidase, is found to be directly associated with 17 genes, including genes promoting growth and development (*GA20OX1*) and three genes associated with chloroplast and photosynthesis (*CP12*, *CAB*, and *PSRP*). *GA20OX1* is a key enzyme in the synthesis of gibberellins, which promotes growth and development. Previous studies suggested that carotenoids and gibberellins are derived from the common precursor GGPP.^41^^,^^42^ The observations are consistent with the function of the carotenoid synthesis pathway in the regulation of plant growth and development.^43^*CP12* encodes a small peptide found in the chloroplast stroma and is coordinately regulated by light with the photosynthetic *GAPDH* and *PRK.*^44^*CAB* is a gene that encodes the chlorophyll a/b-binding protein, which functions as a light receptor and captures and delivers excitation energy to photosystems.^45^ PSRP is a plastid-specific ribosomal protein, acting in light regulation of translation.^46^^,^^47^

### 3.6. Integrative analysis of gene and metabolite modules

**Figure 6:**
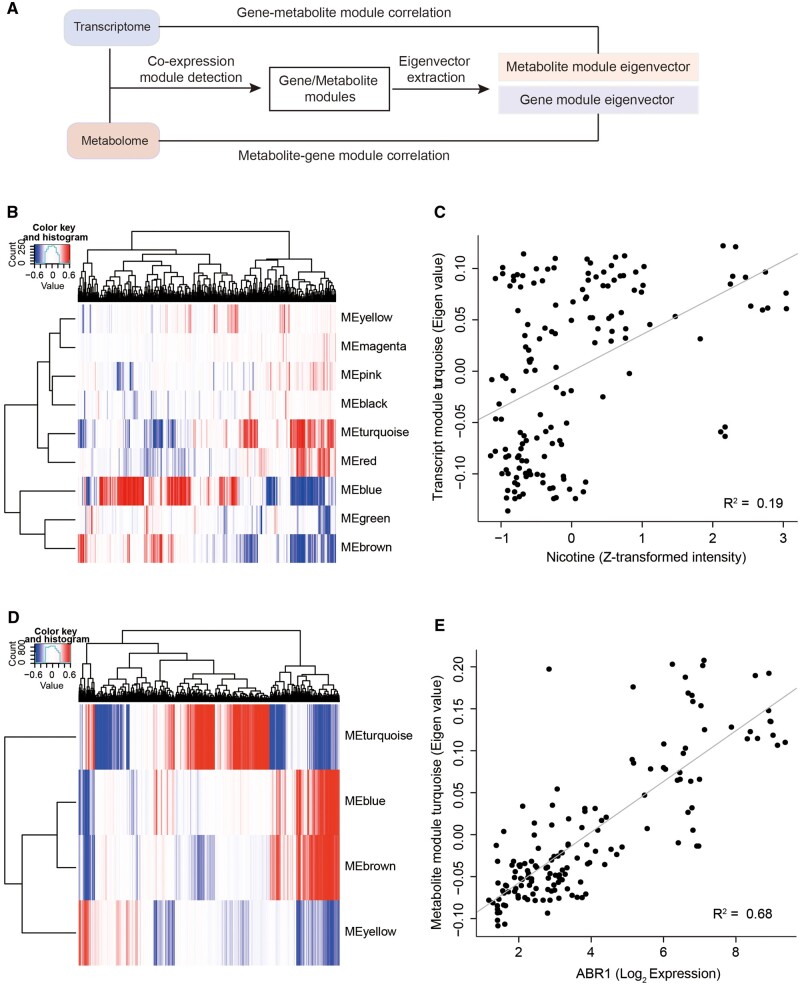
Integrative analysis of transcriptomic and metabolomic data. (A) Diagram showing integration approach of transcriptomic and metabolomic data. (B) Heat map showing metabolites that have high correlation with nine transcript modules (i.e. eigen-value of the modules). Correlation between metabolites and transcript modules was represented using a red–white–blue colour scale: Red represents a positive correlation, green indicates a negative correlation, and white shows no correlation. (C) Scatter plot showing a correlation between transcript turquoise module and nicotine. (D) Heat map showing transcripts that are highly correlated with four metabolite modules. The same colour scheme is used as panel B. (E) Scatter plot showing correlation between metabolite turquoise module and ABR1. Color figures are available at *DNARES* online.

We further performed integrative analysis for identified gene and metabolite co-expression modules using two-way Pearson correlations (Fig. 6A). First, we correlated the abundance of metabolites with the eigenvector (ME) of each gene module to identify the metabolites associated with nine gene modules (Fig. 6B) (Supplementary Table S7A). We found that the ME of the blue gene module shows positively correlated most of the metabolites (*n* = 275), followed by the turquoise gene module (*n *=* *196). In contrast, brown and black gene modules exhibit a negative correlation with metabolites. For example, metabolite nicotine significantly correlated with the ME of the module turquoise (*R*^2^ = 0.19; *P*-value = 1.95 × 10^−8^) (Fig. 6C). The result is reminiscent of our earlier observation that genes in the turquoise module show enrichment in the nicotine synthesis pathway.

We also correlated transcripts with the ME of four metabolite modules: turquoise, blue, brown, and yellow (Fig. 6D). Three MMs (turquoise, blue, and brown) show a significant positive correlation (*P*-value < 0.01) with 2,631, 1,423, and 1,746 transcripts, respectively (Supplementary Table S7B). In contrast, the yellow module shows a negative correlation with 1,844 transcripts. For example, the top statistically significantly correlated gene with the metabolite turquoise module is *ABR1* (*R*^2^ = 0.68, *P*-value = 1.28 × 10^−38^) (Fig. 6E). *ABR1* is an AP2-domain transcription factor that functions as a repressor of ABA response.^48^ ABA is involved in plant growth and primary metabolisms under non-stress conditions, such as carbohydrate and carotenoid metabolisms, which were found to be enriched in the metablite module turquoise.

### 3.7. Identifying potential functional genes by integrative analysis

**Figure 7:**
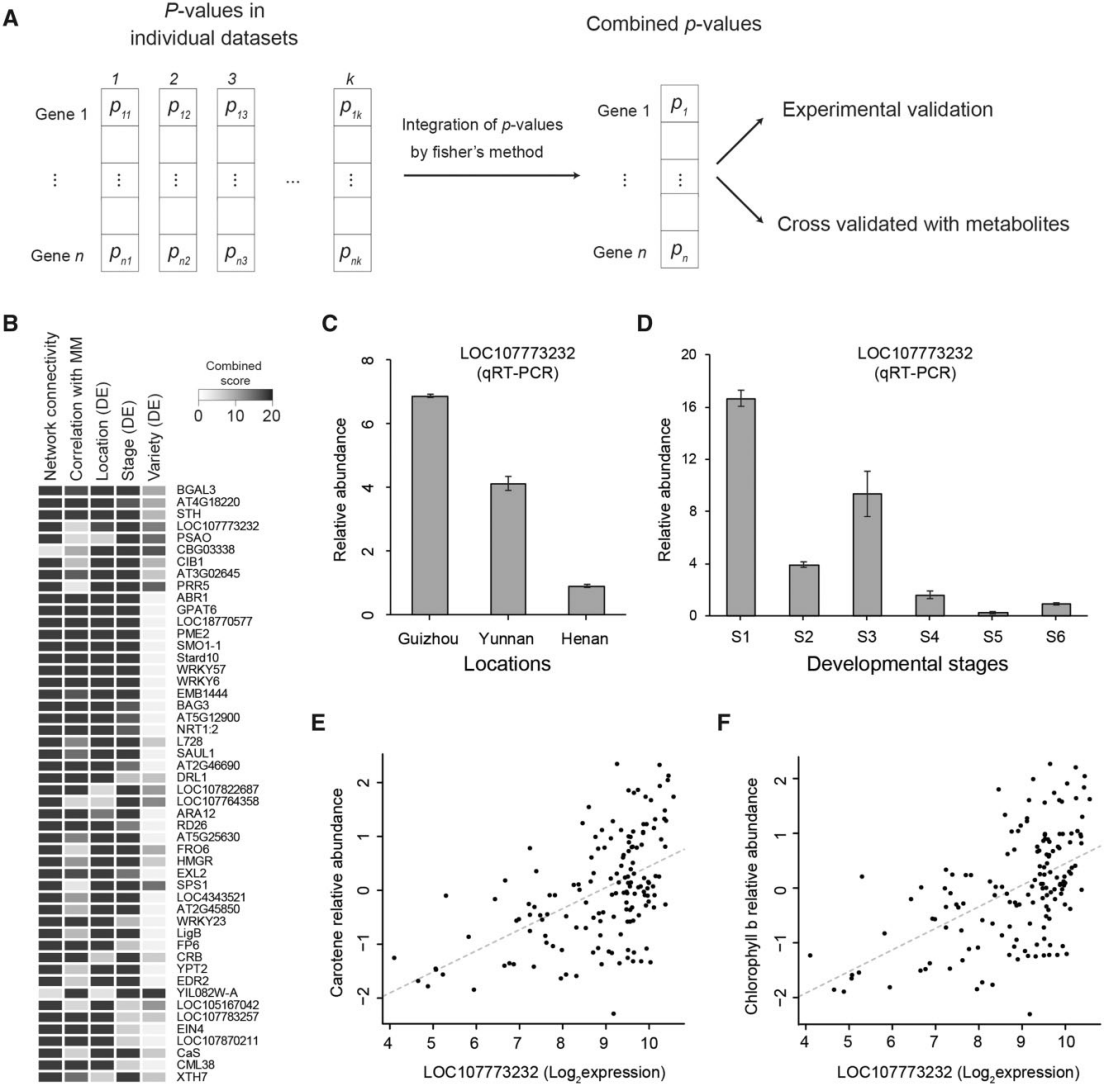
Identifying functional genes by gene prioritization. (A) Diagram showing the prioritization method. Fisher’s combined probability test is used to combine *P*-value from differential expression analyses and network results. (B) Heat map showing individual *P*-values for the top 50 genes ranked by a combined score. The combined score is computed as –log_10_(combined *P*-value). LOC107773232 is ranked as the top 4th most significant genes using the combined score. (C–D) Bar plots showing LOC107773232 expression between three locations and six developmental stages. The expression of LOC107773232 was measured by qRT-PCR. (E–F) Scatter plot showing a high correlation between LOC107773232 expression and the abundance of carotene and chlorophyll b metabolites.

We finally sought to identify novel functional genes in response to genetic and environmental perturbations by considering all results generated by our integrative analysis. To rank genes by its functional importance, we generated a combined score (Fig. 7A) using Fisher’s combined probability test.^27^ The combined score was computed based on five analyses, including the *P* values from DE analyses between three locations, between three varieties, and six developmental stages, the *P*-value of network connectivity, and the *P*-value of the gene correlated with the metabolite module. As expected, most of the top 50 ranked genes are involved in plant development, photosynthesis, and regulators of abiotic stress responses (Fig. 7B). LOC107773232 is ranked at the fourth position in the list, which cannot be captured by individual datasets (Supplementary Fig. S6): ranking as 1448^th^ position in differentially expression analysis between three locations, 273^rd^ position in differentially expression analysis between three varieties, 366th position in differentially expression analysis between six stages, 1062^nd^ position in the network connectivity of gene co-expression analysis, and 168^th^ position in the network connectivity of metabolite co-expression analysis. The gene is a not well-characterized gene whose sequences are homologous to chlorophyll a–b binding protein 40 (CAB40). qPCR confirmed that LOC107773232 shows an elevated expression in Guizhou compared with Yunnan and Henan (Fig. 7C) and a decrease in expression along six developmental stages (Fig. 7D). When correlated metabolites in the carotenoid metabolism pathway, the expression of LOC107773232 was found to be highly correlated with both chlorophyll b (*P*-value = 1.73 × 10^−12^; Fig. 7E) and carotene (*P*-value = 2.79 × 10^−12^; Fig. 7F). Therefore, we surmise that it may be an important regulator involved in the carotenoid metabolism pathway (Fig. 3F).

## 4. Discussion

In this study, we performed systems-level analyses of both transcriptome and metabolome profiled from 54 tobacco samples collected from six developmental stages of three varieties planted in three locations. We identified 3,405 DEGs and 371 DEMs between the three conditions (varieties, locations, and developmental stages), and constructed co-expression modules for both DEGs and DEMs. We further integrated transcript and metabolite co-expression modules using a two-way Pearson correlation. The results provide insights into molecular networks underlying complex agronomic and quality traits in tobacco.

Recently, several studies investigated changes in metabolite abundance in tobacco, and revealed that the most important perturbation in tobacco metabolome is the geographical location.^13^^,^^14^ Consistently, we identified 2,763 DEGs and 291 DEMs between three locations compared with only 941 DEGs and 192 DEMs between three varieties, suggesting that environmental perturbation is a critical factor for transcriptomics data. Those DEGs between locations were mainly enriched in the biological processes of photosynthesis and stress (Supplementary Table S6), which is consistent with the different climatic conditions among three locations: Guizhou, Yunnan, and Henan. For example, most genes enriched in the process of response to light intensity (GO: 0009642) are highly expressed in Guizhou but low in Yunnan, suggesting those genes are negatively regulated by the total sunshine time, but positively respond to total rainfall during the entire growing period (Supplementary Fig. S1). Most DEGs over-represented in the light reaction process (GO: 0019684) show up-regulation in Yunnan, indicating that they were negatively regulated by the temperature because the average daily temperature is typically lower in Yunnan compared with Henan. In summary, DEGs between locations are largely contributed by the climatic conditions. In addition, among 941 DEGs between three varieties, 65% genes shared with DEGs between locations, indicating that part of DEGs between varieties are also contributed by climatic conditions. However, the underlying regulatory mechanisms could be different for several observations because only genes in cluster 3 of DEGs between varieties (Fig. 3C) are significantly enriched in the light harvesting process of photosynthesis (GO: 0009765, Supplementary Table S6), in which genes show higher expression level in Hongda compared with that in Zhongyan. Taken together, the findings provide an opportunity to gain insights into molecular mechanisms underlying response to environmental perturbation in tobacco.

Previous studies only characterized tobacco changes in metabolite or gene abundance under a certain condition, such as developmental stages,^14^^,^^49^^,^^50^ or different locations.^13^ In contrast, we conducted a comprehensive analysis by profiling both transcriptomic and metabolomic across three varieties, three locations, and six developmental stages. Therefore, our analysis not only determined changes in gene and metabolite abundance under a single condition but also characterized changes under the interaction between two conditions. For example, we detected 946 DEGs and 77 DEMs that are influenced by the interaction of variety and developmental stages (Supplementary Tables S3A and B). In addition, our analysis globally examined the alternation of both transcripts and metabolites in a pathway (Fig. 3E and F). Integrative analysis of omics data is still a major challenge in biological research. To address the challenge, mathematical and statistical models are often developed to identify joint systematic variation between transcriptomic and metabolomic data.^51^ In this study, we proposed a novel approach to integrate two omics data by two-way Pearson correlations after detecting co-expression modules: (i) correlating each metabolite with eigenvector of co-expression gene module; and (ii) correlating each gene with eigenvector co-expression metabolite modules. The advantage of our approach compared with other statistical methods is that genes or metabolites in co-expression modules are highly correlated and involved in similar biological functions. In addition, the approach is simple and easy to understand compared with other multi-variate methods, such as the orthogonal partial least squares (O2PLS) model. Despite effectiveness, the correlation analysis suffers from certain limitations. The Pearson correlation typically requires the normal distribution underlying the data. Hence, we should be cautious when using data that do not follow the normal distribution, such as genotypic data. Moreover, correlation cannot be taken to imply causation although there is a very strong association between two variables. Developing more sophisticated statistical approaches will be needed to infer the causality, such as structural equation modeling.

Over the past decades, traditional genetics and breeding approaches using quantitative trait locus mapping and DNA marker-assisted breeding have been widely used to improve agronomic and quality traits.^52–54^ Although many loci controlling these traits have been identified,^54^ they provided little information on molecular networks linking genetic loci to specific traits. That is, how variant(s) at the DNA level are translated to a phenotype through genetic information flow. In contrast, in this study, we globally examined changes at both transcript and metabolite levels and constructed molecular networks to probe the potential molecular networks underlying a trait. The advantage of this systems-biology approach is that it allows us to analyse molecular interactions within a single omics layer and *trans*-omics layers.

We identified DEGs and DEMs as well as their co-expression modules. Some of the modules can be potentially linked to nicotine synthesis and carotenoid synthesis pathways (Fig. 5). However, further experimental validation is still needed using additional platforms in addition to large-scale omics profiling. For example, DEGs can be validated by RT-PCR and DEMs can be validated by targeted metabolomics using standard compounds. If any important genetic variants within candidate DEGs are observed between different varieties, we could perform some functional assays using gene-editing technologies, such as CRISPR technology.

In this study, although we integrate transcripts with metabolites, proteins are typically considered to be closer to metabolites and classical phenotypes than transcripts. Therefore, information at the protein level could provide intermediate information between transcripts and metabolites. In the near future, we will profile whole proteome data using high-resolution MS. In addition, we will only focus on enzymes that are directly interacted with metabolites in metabolic networks since only enzymes directly interact with metabolites. For network construction, we will use both data-driven and knowledge-based approaches to integrate multi-omics data. Moreover, single-cell RNA sequencing and single cell-type proteomics are also emerging. With these new technologies, we could potentially identify the major cell types involved in molecular networks in response to genetic and environmental perturbations, and developmental processes in tobacco.

## 5. Conclusion

In conclusion, we performed an integrative analysis of both transcriptomic and metabolomic data and identified molecular networks that are involved in agronomic and quality traits in tobacco. This study underscores the importance of integrative analyses in elucidating regulatory networks underlying complex traits. The findings in this study could improve the tobacco quality traits by comprehensive understanding of molecular mechanisms of how genetic and environmental perturbations influence developmental processes and formation of complex phenotypes.

## Supplementary data

Supplementary data are available at DNARES online.

## Accession numbers

BCWF01000001-BCWF01000044

## Funding

This study was supported by the Project of ENCODE of Tobacco Genome (No.110201401012 (JY-12), No.110201601033 (JY-07)), and Natural Science Foundation of Zhejiang Province (LY20C150004).

## Conflict of interest

None declared.

## Supplementary Material

dsaa006_Supplementary_Data
