# Supplementary material for: Integrating transcriptome and metabolome reveals molecular networks involved in genetic and environmental variation in tobacco
Source: DNA Res. 2020 Apr 23;27(2):dsaa006. doi: 10.1093/dnares/dsaa006 (PMC7320822; doi:10.1093/dnares/dsaa006)
Supplement: dsaa006_Supplementary_Data [file dsaa006_supplementary_data.zip › Supplemental Materials_v0.0.2.doc]

Supplemental Figures for

**Integrating Transcriptome and Metabolome Reveals Molecular Networks involved in Genetic, Environmental and Developmental Variation in Tobacco**

Pingping Liu^1,&^, Jie Luo^2,&,*^, Qingxia Zheng^1^, Qiansi Chen^1^, Niu Zhai^1^, Shengchun Xu^2^, Yalong Xu^1^, Lifeng Jin^1^, Guoyun Xu^1^, Xin Lu^3^, Guowang Xu^3^, Gangjun Wang^2^, Jianfeng Shao^2^, Hai‐Ming Xu^4^, Peijian Cao^1^, Huina Zhou^1,*^, and Xusheng Wang^5,*^

**Supplemental Figures**

Figure S1. Climatic data from three locations: Guizhou, Henan, and Yunnan.

Figure S2. Venn diagram comparing significantly differentially expressed genes and metabolites.

Figure S3. PCA plots of the 54 samples for six stages on the top of three locations.

Figure S4. Differentially expressed genes validated by quantitative real-time PCR (qPCR).

Figure S5. Expression patterns of two key genes and metabolites from both nicotine synthesis and carotenoid synthesis pathways.

Figure S6. Ranking of LOC107773232 in single layer of the data and combined method.

**Supplemental Tables**

Table S1: Samples for transcriptome and metabolome.

Table S2: Metabolome profiling from 151 Tobacco samples.

Table S3A: Differentially expressed genes detected under three locations, three varieties, six stages, as well as their interactions.

Table S3B: Differentially altered metabolites detected under three locations, three varieties, six stages, as well as their interactions.

Table S4A: Co-expressed gene modules.

Table S4B: Co-expressed metabolite modules.

Table S5: Sizes of gene and metabolite co-expressed modules.

Table S6: Enrichment analysis of differentially expressed genes.

Table S7A: Correlation between metabolites and co-expressed gene modules (kME).

Table S7B: Correlation between transcripts and co-expressed metabolite modules (kME).

Table S8. Genes and metabolites in our curated pathways.

Table S9. Primers of 20 genes for qPCR validation.

**Figure S1. Climatic data from three locations: Guizhou, Henan, and Yunnan**. Three climatic factors were measured and daily averaged, including temperature, sunshine time, and Rainfall. Three stages (i.e. root extending, vigorous growth, mature) were examined at each climatic factor.

**Figure S3. PCA plots of the 54 samples for six stages on the top of three locations.** For each location, samples from each stage were grouped by each dash oval. Samples of the stage 1 from both Henan and Guizhou was not grouped by oval because of too sparse while they are well separated.

**Figure S3. Venn diagram comparing significantly differentially expressed genes and metabolites**. The differentially expressed genes are detected in the three single-factor comparisons of varieties, locations, and developmental stages.

**Figure S4.** **Differentially expressed genes validated by quantitative real-time PCR (qPCR).** (A-B) Heat map showing expression patterns across three locations and six developmental stages by both microarray and qPCR analyses. Y, G, and H denote Yunnan, Guizhou, and Henan, respectively. The expression of each gene is Z-score transformed. (C-D) qPCR showing a consistent trend of the expression of CAB16 between three locations and six developmental stages. Three replicates were used in both qPCR and Microarray analyses.

**Figure S5. Expression patterns of two key genes and metabolites from both nicotine synthesis and carotenoid synthesis pathways.** For each gene and metabolite, three varieties, three locations, and six stages are shown.

**Figure S6. Ranking of *LOC107773232* in single layer of the data as well as in the integrative analysis.**
